# Prevalence of Past and Reactivated Viral Infections and Efficacy of Cyclosporine A as Monotherapy or in Combination in Patients with Psoriatic Arthritis—Synergy Study: A Longitudinal Observational Study

**DOI:** 10.1155/2014/941767

**Published:** 2014-04-03

**Authors:** Delia Colombo, Sergio Chimenti, Paolo Grossi, Antonio Marchesoni, Sergio Di Nuzzo, Vito Griseta, Anna Gargiulo, Aurora Parodi, Lucia Simoni, Gilberto Bellia

**Affiliations:** ^1^Novartis Farma Italia, Origgio (Va), Italy; ^2^Policlinico Tor Vergata, Rome, Italy; ^3^Ospedale di Circolo e Fondazione Macchi, Varese, Italy; ^4^Istituto Ortopedico Pini, Milan, Italy; ^5^Azienda Ospedaliero-Universitaria, Parma, Italy; ^6^Ospedale Regionale F. Miulli, Acquaviva delle Fonti (BA), Italy; ^7^Azienda Ospedaliera Sant'Anna e San Sebastiano, Caserta, Italy; ^8^Ospedale San Martino, Genoa, Italy; ^9^MediData srl, Modena, Italy

## Abstract

We have prospectively evaluated psoriatic arthritis (PsA) patients for (1) seropositivity for former viral infections and seroconversion and (2) efficacy of cyclosporine A (CsA) alone or in combination with other immunosuppressants in a time period of 12 months. Screening included HBV antibodies and antigens, HCV antibodies and RNA, HSV 1-2, HZV, EBV, and CMV IgG, and IgM, HHV-6 DNA, and HIV 1-2 antibodies. PsA was evaluated by the Psoriasis Area Severity Index (PASI), the Bath Ankylosing Spondylitis Disease Activity Index (BASDAI), and the Visual Analogue Scale (VAS). At baseline, 126 (56%) out of 225 evaluable patients had 2 or more seropositivities indicative of former infections, and 31 patients (13.8%) presented seropositivity for HCV, HBV, HSV-1 and -2, HHV-6, EBV, or parvovirus infection; one of them, positive for HBAg, was treated with lamivudine, while the remaining 30 received no specific treatment. None of the 31 patients developed virus reactivation. A reduction (*P* < 0.001) of PASI, BASDAI, and VAS scores was observed at 6 and 12 months. The treatment of PsA with CsA as monotherapy or in combination was safe and effective. *In vitro* experiments and clinical findings, including those from our study, suggest that CsA as monotherapy or in combination with biologics might be the treatment of choice in PsA HCV-positive patients.

## 1. Introduction

Psoriatic arthritis (PsA) is a disabling complication which occurs in 6% to 39% of psoriatic patients [1, 2]. The clinical picture is characterized by skin and nail psoriasis associated with heterogeneous spondyloarthropathy, usually manifesting as synovitis, enthesitis, dactylitis, and spondylitis [3]. In most patients, the skin disease precedes musculoskeletal involvement. Obesity is a risk factor for the development of PsA in patients with psoriasis [4]. Its early recognition, diagnosis, and treatment can relieve pain and inflammation and, possibly, help prevent progressive joint involvement and damage [3]. Over the past years, advances have been made in the understanding of genetic and molecular mechanisms of PsA and its management [3].

Patients with PsA are often treated with systemic immunosuppressant agents (e.g., steroids, methotrexate, cyclosporine A, biologic drugs), which might increase the risk of reactivation of past infections or the acquisition of new infections. Such infections could possibly be life-threatening. Despite the influence of these treatments on the development of infections, data addressing this issue are scant.

Aims of this study were to evaluate, in a population of patients with PsA, (1) seropositivity for former viral infections or suggestive for acute viral infection, (2) efficacy of cyclosporine A (CsA) administered in routine clinical practice alone or in combination with other immunosuppressants in a time period of 12 months, and (3) the occurrence of adverse events during the study period.

## 2. Patients and Methods

The study was approved by the local Ethics Committee at each participating center and signed informed consent was obtained from participating patients. This was a cross-sectional and longitudinal study (12 months), carried out in 238 consecutive patients with diagnosis of PsA performed within 8 years from baseline, treated for at least 3 months with CsA as monotherapy or in combination with one or more systemic drugs. The diagnosis of PsA was performed according to the CASPAR criteria [5]. Psoriasis was evaluated by the Psoriasis Area Severity Index (PASI) [6]. PsA was evaluated based on the joints involved, the patients' and the physicians' global assessment of the disease using the Visual Analogue Scale (VAS) [7]. The Bath Ankylosing Spondylitis Disease Activity Index (BASDAI) [8] was used to evaluate inflammation of the spine. Baseline screening for latent and acute viral infections included hepatitis B virus (HBV) antibodies (anti-HBs and anti-HBc) and surface antigen, hepatitis C virus (HCV) antibodies and RNA, herpes simplex virus 1-2 (HSV 1-2) IgG and IgM, herpes zoster virus (HZV) IgG and IgM, human herpes virus 6 (HHV-6) DNA, Epstein-Barr virus (EBV) IgG and IgM, human immunodeficiency virus 1-2 (HIV 1-2) antibodies, cytomegalovirus (CMV) IgG and IgM, and parvovirus B19 IgG and IgM. Follow-up visits were performed at 6 and 12 months to investigate new pathologies and treatments, evolution of PsA (PASI, VAS, and BASDAI), and occurrence of adverse events.

Data are reported as mean ± SD, median (IQ_25–75_), or percentage, as appropriate. Changes in the PASI and BASDAI values at selected time points were compared using the paired* t*-test.

## 3. Results

### 3.1. Patients and Treatments

Two hundred thirty-eight patients with PsA were screened in 24 centers. Thirteen patients not fulfilling the inclusion criteria were excluded from the analysis which, therefore, was conducted on 225 patients at baseline. The number of patients evaluable at 6- and 12-month followups was 177 and 174, respectively. Eleven patients (4.9%) had been vaccinated for HB and were excluded from baseline and follow-up analysis for HBV seroconversion.

Demographic and clinical characteristics at baseline are reported in Table 1. The most common type of psoriasis was* psoriasis vulgaris* (plaque psoriasis). As expected, PsA was equally distributed among males and females, and, on average, the diagnosis of psoriasis required less time than the diagnosis of PsA; the time between psoriasis and PsA diagnoses was 9.3 years (±13.6), on average. Median time (IQ_25–75_) from the diagnosis of the disease to the first systemic treatment for psoriasis or PsA was 4 years (IQ_25–75_ 1–13). One hundred five patients (46.7%) had comorbidities; cardiovascular comorbidities were observed in 62 patients (27.6%).

Systemic, topic, and other treatments at baseline and follow-up visits are reported in Table 2. At baseline, all patients were treated with oral CsA for at least 3 months, 48.9% as systemic monotherapy, 16% in combination with methotrexate, and 10.2% in combination with biologic agents; 78% of patients received also topic treatments. During the study period there was a modest decrease in the use of CsA as both monotherapy and in combination with methotrexate, while a significant increase was observed in the combination CsA/biologic agents (Table 2). The study period was also characterized by a decrease in the frequency of topic therapy (from 78% at baseline to 59% at the end of the study).

### 3.2. Prevalence of Latent Viral Infections

**Figure 1:**
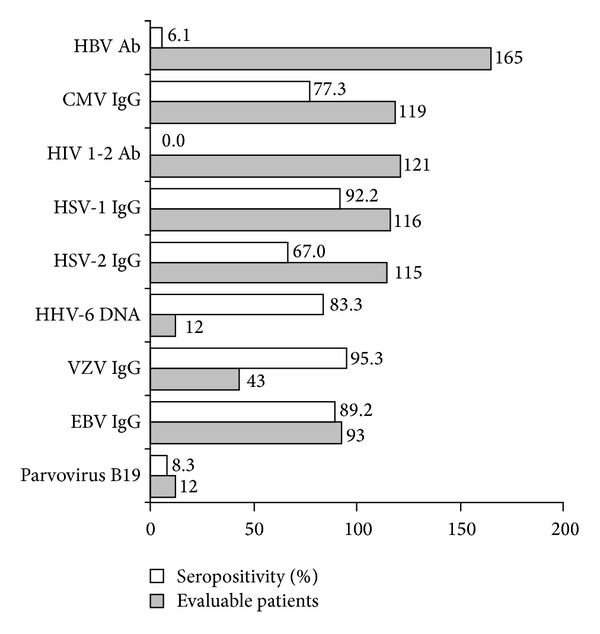
Chronic viral seropositivity (former viral infection) at baseline (%, white bars) in evaluable patients (number, grey bars).

At baseline, 56% of evaluable patients had 2 or more seropositivities indicative of former viral infections (Figure 1). The most frequent were VZV (95.3%), HSV-1 (92.2%), EBV (89.2%), CMV (77%), and HSV-2 (67%). No subject showed HIV antibodies. None of these patients experienced reactivation or new onset viral infections or other infectious diseases during the following 12 months. A linear relationship was observed between the number of executed tests and infections (Pearson's correlation coefficient = 0.80865, *P* < 0.0001).

### 3.3. Prevalence of Critical Viral Infections

At baseline, 31 patients (13.8%) presented seropositivity for HCV, HBV, HSV-1 and -2, HHV-6, EBV, and parvovirus B19 (Table 3). One of them, positive for HBsAg, was treated with lamivudine, while the remaining 30 received no specific treatment or prophylaxis. In all these patients the viral infection was asymptomatic.

During the following 12 months all 31 patients continued their immunosuppressive treatment of PsA and none developed virus reactivation (Table 3). Two HCV-RNA-positive patients and one HBsAg-positive patient were negative after 12 months.

### 3.4. Evolution of Psoriasis and PsA during the Study Period

**Figure 2:**
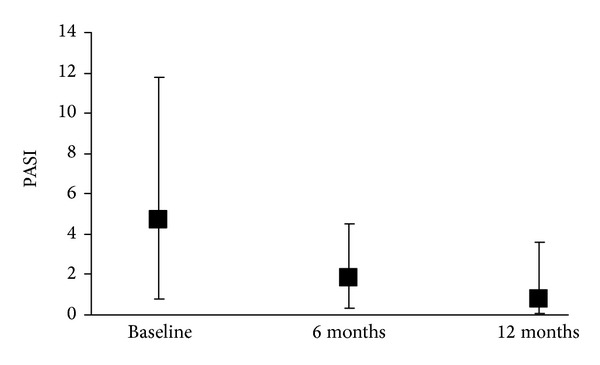
Psoriasis Area Severity Index (PASI) median values and 25°–75° percentiles during the study period in 153 patients evaluable at baseline and at follow-up visits.

Evaluation of psoriasis and PsA during the study period was one of the secondary objectives of the study. PASI, VAS, and BASDAI were assessed at baseline, 6 and 12 months. A progressive and significant (*P* < 0.001 versus baseline) reduction of PASI was observed at 6 and 12 months (Figure 2), indicating an improvement of psoriasis during the study period.

Similarly, there was a decrease in the global activity of PsA during the study, shown by a decrease in both VAS and BASDAI (*P* < 0.0001 versus baseline) (Table 4), indicating an improvement in the musculoskeletal involvement of PsA.

### 3.5. Adverse Events

Eighteen adverse events were reported in 15 patients (Table 5). These adverse events were considered to be correlated with the treatment in 10 cases (to CsA in 9 cases and to infliximab in 1 case). One serious adverse event occurred (syncope); the patient fully recovered. No deaths occurred.

## 4. Discussion

### 4.1. Chronic and Acute Viral Seropositivity

PsA is an uncommon disease affecting 0.3–1% of the general population [1, 9]. Therefore, the strength of this study is the high number of patients recruited and the execution of viral serologic tests not routinely performed in clinical practice. The primary endpoint of the study was to evaluate the prevalence of latent and acute viral infections in patients with PsA. Our data confirm previous observations from the literature indicating that patients with PsA are exposed to multiple viruses with increased prevalence compared to the normal population (Figure 1).

However, the most interesting finding of our study was that 31 patients with serologic findings suggestive of critical viral infections, despite immunosuppressive therapy, did not develop any virus-related clinical symptoms [10]. These patients were all chronically treated with CsA as monotherapy or in combination with other systemic immunosuppressants (Table 3). Although old and new immunosuppressive agents have the potential to induce virus reactivation, data from the literature are very limited in PsA, and the guidelines for the treatment of PsA do not provide therapeutic indications for patients with acute or latent viral infections [11]. However, the possibility that PsA patients also have viral infections is not negligible, and the administration of immunosuppressants to patients with moderate or severe forms can be necessary even in the presence of latent viral infections.

A few considerations can be made for HCV-positive PsA patients. The literature reports that systemic administration of corticosteroids in liver transplant recipients can increase HCV infectivity and exacerbate underlying viral infections [12, 13]; therefore, corticosteroids should probably be avoided in PsA HCV-positive patients. On the contrary,* in vitro* studies have shown that CsA inhibits HCV replication by inhibiting cyclophilin B [14–16]. Moreover, patients treated with CsA after liver transplantation have lower HCV titers and reduced frequency of deaths or graft losses than patients treated with tacrolimus [17, 18].

Few cases of patients with PsA and HCV treated with CsA are reported in the literature. Miura et al. [19] described 4 patients with dermatologic diseases and HCV infection treated with CsA: in 3 cases, CsA was safe and it reduced HCV-RNA load and serum liver enzymes; the fourth patient showed a modest increase in alanine aminotransferases (ALT) levels but no change in blood HCV-RNA load. Galeazzi et al. [20] used CsA in 20 patients with rheumatologic disorders (4 with PsA) and concomitant HCV infection; they reported that HCV RNA load decreased significantly after 6 months. Giannitti et al. [21] treated 7 patients affected by rheumatoid arthritis and HCV infection with CsA and anti-TNF-alpha in combination and found that HCV RNA decreased significantly.

Our results support these earlier observations. An interesting and unexpected finding of our study was that two HCV- RNA-positive patients at baseline evaluation were negative after 12 months of treatment with CsA.

### 4.2. Efficacy during Routine Clinical Practice

During the 12-month period of the study, we observed a progressive improvement in our patients' cutaneous and musculoskeletal clinical picture. We hypothesize the improvement to have been related in part to the progressive therapeutic effect of CsA and, in part, to the increased number of patients who received biologics in combination with CsA.

## 5. Conclusions

Our study shows that PsA patients have a higher prevalence of viral seropositivity than the healthy population. Systematic screening for infections could improve identification of PsA patients at risk of developing active infections. In our patients, the treatment of PsA with CsA as monotherapy or in combination was effective and safe.* In vitro* experiments and clinical findings [22], including those obtained in our study, suggest that CsA as monotherapy or in combination with biologics might be the treatment of choice in HCV-positive patients if PsA needs systemic treatment, but caution and close laboratory and clinical monitoring are necessary prior to and during treatment.

## Figures and Tables

**Table 1:** Demographic and clinical characteristics at baseline.

Sex, number (%)	
Male	121 (54)
Female	104 (46)

Age (years, mean ± SD)	
Male	48.9 ± 12.8
Female	50.8 ± 12.5

Treating physician, number (%)	
Dermatologist	166 (73.8)
Rheumatologist	52 (23.1)
Both	4 (1.8)

Age at diagnosis of psoriasis (years, mean ± SD)	38.3 ± 15.6

Age at diagnosis of psoriatic arthritis (years, mean ± SD)	47.6 ± 12.4

PASI, median (IQ_25–75_)	4.7 (0.8–11.8)

BASDAI, median (IQ_25–75_)	40.8 (23.5–56.8)

Classification according to joint involvement, %^1^	
Monoarticular	19 (8.4)
Oligoarticular	81 (36.0)
Polyarticular	92 (41.0)
Spondylotic	36 (16.0)
Enthesic	40 (18)

Comorbidities, no. (%)	
Cardiovascular	62 (27.6)
Diabetes mellitus type 2	13 (5.8)
Diabetes mellitus type 1	4 (1.8)
Renal disease	5 (2.2)
Cancer	1 (0.4)
Hepatitis B	1 (0.4)
Other	49 (21.8)

Data refer to 225 patients except for BASDAI (61 evaluable patients). PASI: Psoriasis Area Severity Index; BASDAI: Bath Ankylosing Spondylitis Disease Activity Index. ^1^Patients can be classified in more than one group.

**Table 2:** Description of treatments at baseline and at the end of study.

	Baseline (number = 225)	From baseline to 12-month followup (number = 174)
Systemic treatments, number (%)		
Cyclosporine	110 (48.9)	70 (40.2)
Cyclosporine + methotrexate	36 (16.0)	26 (14.9)
Cyclosporine + biologic	23 (10.2)	38 (21.8)
Cyclosporine + other systemic treatments	45 (20.0)	27 (15.5)
m.d.	11 (4.9)	13 (7.5)

Topic treatments, no. (%)^1^		
Total number of patients treated	175 (77.8)	103 (59.2)
No topic treatment	47 (20.9)	68 (39.1)
m.d.	3 (1.3)	3 (1.7)
Corticosteroids	157 (69.8)	77 (44.3)
Vit D3 derivatives	98 (43.6)	53 (30.5)
Retinoids	27 (12.0)	8 (4.6)
PUVA	5 (2.2)	2 (1.1)
UVB	8 (3.6)	4 (2.3)
Laser	1 (0.4)	1 (0.6)
Other	15 (6.7)	21 (12.1)

Intra-articular injection	5 (2.2)	6 (3.4)

^1^Patients might have been treated with more than one topic treatment. m.d.: missing data.

**Table 3:** Description of patients with acute viral seropositivity.

	Basal	Number of patients who developed a virus-correlated disease	Treatment of viral seropositivity	Systemic treatment of psoriatic arthritis during the study
HCV RNA (*n* = 17)	7 pos	0	No	5 CsA; 1 CsA + steroid + methotrexate; 1 CsA + biologic
HBsAg (*n* = 188)	6 pos	0	1 pt (lamivudine)	3 CsA; 2 CsA + MTX; 1 CsA + steroid + MTX
HSV-1 IgM (*n* = 115)	3 pos	0	No	1 CsA; 1 CsA + steroid; 1 CsA + steroid + MTX
HSV-2 IgM (*n* = 114)	3 pos	0	No	1 CsA; 1 CsA + steroid; 1 CsA + steroid + MTX
HHV-6 DNA (*n* = 12)	10 pos	0	No	7 CsA; 3 CsA + biologic
EBV IgM (*n* = 99)	1 pos	0	No	1 CsA + steroid + biologic
Parvovirus B59 IgM (*n* = 12)	1 pos	0	No	1 CsA

CsA: cyclosporine A; MTX: methotrexate.

**Table 4:** Clinical assessment of psoriatic arthritis (PsA) and spondylitis in patients evaluable at baseline and at follow-up visits (median and IQR).

	Baseline	6 months	12 months
VAS, patients' global assessment (no. = 144)	50 (30–71.5)	30 (12.5–50)*	20 (10–43)*
VAS, physicians' global assessment (no. = 147)	40 (20–60)	20 (5–44)*	10 (5–30)*
BASDAI (no. = 61)	40.5 (24–60)	21 (7–38)*	20 (5–38)*

Patients' and physicians' global assessment was performed using the Visual Analogue Scale (VAS). *P < 0.0001 versus baseline.

**Table 5:** List of adverse events.

Adverse event	Severity	Drug-related	Systemic treatment of psoriasis or psoriatic arthritis concomitant to adverse event
Increased creatinine and increased serum cholesterol	Mild	Yes, cyclosporine	Cyclosporine
Increased creatinine	Mild	Yes, cyclosporine	Cyclosporine, sulfasalazine
Gingival hyperplasia and gingival bleeding	Mild	Yes, cyclosporine	None
Tremor	Mild	Yes, cyclosporine	Cyclosporine
Burning urination	Mild	No	Cyclosporine, methotrexate, quinolones
Urethritis	Mild	No	Fans
Urethritis (*Proteus mirabilis*)	Mild	No	Fans
Hypertension	Moderate	Yes, cyclosporine	Etanercept, cyclosporine
Increased creatinine	Moderate	Yes, cyclosporine	Cyclosporine
Fatigue, stomatitis, gastrointestinal disorders, and vomiting	Moderate	Yes, cyclosporine	Cyclosporine
Nausea and vomiting	Moderate	Yes, cyclosporine	Cyclosporine, methotrexate
Hypertension, altered liver Function tests	Moderate	Yes, cyclosporine	Cyclosporine
Fever, sore throat, hyposmia, hypoacusia	Moderate	No	Methotrexate
Postmicturition syncope	Moderate	No	Cyclosporine
Presyncopal episode preceded by autonomic problems (sweating, dizziness, nausea) after several episodes of diarrhea (enteritis)	Moderate	No	Cyclosporine
Renal colic	Moderate	No	Cyclosporine, leflunomide
Syncopal episode	Serious	UKN	Cyclosporine, methotrexate
Outcomes of central vein occlusion	UKN	Yes, infliximab	Infliximab, methotrexate, cyclosporine
